# Supplementary material for: Content-rich biological network constructed by mining PubMed abstracts
Source: BMC Bioinformatics. 2004 Oct 8;5:147. doi: 10.1186/1471-2105-5-147 (PMC528731; doi:10.1186/1471-2105-5-147)
Supplement: Additional File 2 — The original results of the above study (non-essential files are deleted to keep the file size under the limit set by BMC bioinformatics). [file 1471-2105-5-147-S2.bz2 › chilibotAdditionalFile2/dip50/341ID8076595E4256/html/TNFSF4_TNFRSF4.html]

 


 **TNFSF4** and **TNFRSF4** 
  
Found 114 abstracts in PubMed, retrieved 50.  
 

 What does Google say? 
 PDF only 
| .edu only 

---

**Interactive relationship** (e.g. stimulation, inhibition, etc)

**Inhibitory relationship**- Reverse transcriptase PCR analysis showed that expression of RANTES mRNA was induced after incubation with soluble OX40  [ **TNFRSF4** ]  and this induction was inhibited by anti gp34  [ **TNFSF4** ]  mAb.  Ref: 12161277 Immunol Lett, 2002
- Coculture with MMCE gp34  [ **TNFSF4** ]  inhibited the apoptosis of Kit225 OX40  [ **TNFRSF4** ] , but Kit225 del OX40  [ **TNFRSF4** ]  apoptosis was hardly affected.  Ref: 12416737 Int J Hematol, 2002
- Third, animals were treated with the fusion protein OX40  [ **TNFRSF4** ]  Ig which interferes with the OX40  [ **TNFRSF4** ]  OX40L  [ **TNFSF4** ]  interaction and thereby inhibits the intestinal manifestations of acute GvHD.  Ref: 11686535 Int J Colorectal Dis, 2001
- OX40  [ **TNFRSF4** ]  IgG fusion proteins act as decoys and inhibit T cell costimulation and extravasation induced by OX40  [ **TNFRSF4** ]  ligand  [ **TNFSF4** ]  expressing antigen presenting cells APC and vascular endothelial cells, respectively.  Ref: 12223520 J Leukoc Biol, 2002
**Stimulatory relationship**- HUVECs expressing high levels of gp34  [ **TNFSF4** ]  were stimulated with recombinant soluble OX40  [ **TNFRSF4** ]  or mock control and subjected to analysis using cDNA expression arrays.  Ref: 12161277 Immunol Lett, 2002
- OX40  [ **TNFRSF4** ]  is a member of the tumor necrosis factor  [ **TNFSF4** ]  TNF receptor superfamily and known to be an important costimulatory molecule expressed on activated T cells.  Ref: 11435553 J Virol, 2001
- The coexpression of OX40  [ **TNFRSF4** ]  and IL 2Ralpha on activated CD4 T cells was previously reported. CONCLUSION OX40  [ **TNFRSF4** ] , expressed in a fraction of activated CD4 T cells, may be upregulated in thymic tissues adjacent to GC and thymoma in MG, and OX40  [ **TNFRSF4** ]  may interact with OX40L  [ **TNFSF4** ]  in GC to enhance anti acetylcholine receptor antibody production in MG.  Ref: 11071109 Acta Neurol Scand, 2000
- gp34  [ **TNFSF4** ] , which we previously cloned, is a ligand of OX40  [ **TNFRSF4** ]  CD134  [ **TNFRSF4** ] , a costimulatory molecule involved in T cell activation.  Ref: 11346458 Jpn J Cancer Res, 2001
- The transferred OX40L  [ **TNFSF4** ]  on normal CD4 T cells was functionally active as they stimulated latent HIV 1 infected cells to produce viral proteins via OX40  [ **TNFRSF4** ]  signaling.  Ref: 11441094 J Immunol, 2001
- Furthermore, enhanced HIV 1 transcription in gp34  [ **TNFSF4** ]  stimulated ACH 2 OX40  [ **TNFRSF4** ]  cells was dependent on the kappa B site of the HIV 1 long terminal repeat, and the OX40  [ **TNFRSF4** ]  gp34  [ **TNFSF4** ]  interaction activated NF kappa B consisting of p50 and p65 subunits.  Ref: 11435553 J Virol, 2001
**Neutral relationship**- Thus, OX40  [ **TNFRSF4** ]  OX40L  [ **TNFSF4** ]  interactions are pivotal to the efficiency of recall responses regulated by memory Th2 cells.  Ref: 12860930 J Exp MedJ Exp Med, 2001
- Interaction of OX40  [ **TNFRSF4** ]  CD134  [ **TNFRSF4** ]  and its ligand, OX40L  [ **TNFSF4** ] , has been implicated in T cell activation and cell migration.  Ref: 12794142 J Immunol, 2003
- In already sensitized animals, blocking OX40  [ **TNFRSF4** ]  OX40L  [ **TNFSF4** ]  interactions at the time of inhalation of aerosolized antigen suppressed memory effector accumulation in lung draining lymph nodes and lung, and prevented eosinophilia, airway hyperreactivity, mucus secretion, and Th2 cyto kine production.  Ref: 12860930 J Exp MedJ Exp Med, 2003
- Interaction of OX40L  [ **TNFSF4** ]  with its receptor OX40  [ **TNFRSF4** ]  is thought to be important in T cell activation through T cell antigen presenting cell interaction.  Ref: 11069062 Eur J Immunol, 2000
- Contribution of OX40  [ **TNFRSF4** ]  OX40  [ **TNFRSF4** ]  ligand  [ **TNFSF4** ]  interaction to the pathogenesis of rheumatoid arthritis.  Ref: 11069062 Eur J Immunol, 2000
- Constitutive OX40  [ **TNFRSF4** ]  OX40  [ **TNFRSF4** ]  ligand  [ **TNFSF4** ]  interaction induces autoimmune like diseases.  Ref: 12370402 J Immunol, 2002
- The interaction between OX40  [ **TNFRSF4** ]  and OX40  [ **TNFRSF4** ]  ligand  [ **TNFSF4** ]  OX40L  [ **TNFSF4** ]  is suggested to provide T cells with an effective costimulatory signals during T cell APC interaction.  Ref: 12370402 J Immunol, 2002
- Blockade of OX40  [ **TNFRSF4** ]  OX40L  [ **TNFSF4** ]  interaction in the recipient RAG2 deficient mice completely prevented disease development.  Ref: 12370402 J Immunol, 2002
- Through interactions with OX40  [ **TNFRSF4** ]  ligand  [ **TNFSF4** ] , OX40  [ **TNFRSF4** ]  delivers potent costimulatory signals to T cells.  Ref: 11470287 J Immunol Methods, 2001
- OX40  [ **TNFRSF4** ]  OX40  [ **TNFRSF4** ]  ligand  [ **TNFSF4** ]  OX40L  [ **TNFSF4** ]  proteins play critical roles in the T cell B cell and T cell dendritic cell interactions.  Ref: 11441094 J Immunol, 2001
- Using these mice, we demonstrate here the critical involvement of the OX40L  [ **TNFSF4** ]  OX40  [ **TNFRSF4** ]  interaction during the T cell priming events in the occurrence of experimental autoimmune encephalomyelitis EAE.  Ref: 11509650 J Immunol, 2001
- Recently, several groups have reduced clinical signs of autoimmunity in animal models by blocking the OX40  [ **TNFRSF4** ]  OX40  [ **TNFRSF4** ]  ligand  [ **TNFSF4** ]  interaction or depleting OX40  [ **TNFRSF4** ]  T cells.  Ref: 11929124 Trends Immunol, 2002
- These results suggest that the OX40  [ **TNFRSF4** ]  OX40L  [ **TNFSF4** ]  interaction plays a critical role for the migration of pathogenic T cells into the CNS in the pathogenesis of EAE.  Ref: 11160262 J Immunol, 2001
- The role of OX40  [ **TNFRSF4** ]  ligand  [ **TNFSF4** ]  interactions in the development of the Th2 response to the gastrointestinal nematode parasite Heligmosomoides polygyrus.  Ref: 12496423 J Immunol, 2003
- These studies suggest an important role for OX40  [ **TNFRSF4** ]  OX40L  [ **TNFSF4** ]  interactions in specifically promoting IL 4 production, as well as associated IgE elevations, in Th2 responses to H. polygyrus.  Ref: 12496423 J Immunol, 2003
- However, in vivo blockade of OX40  [ **TNFRSF4** ]  OX40L  [ **TNFSF4** ]  interactions reduced GVHD mortality induced by either signal transducer and activator of transcription 6 Stat 6 Th2 defective or Stat 4 Th1 defective major histocompatibility complex MHC disparate splenocytes, indicating that the GVHD ameliorating effects did not require Stat 4 or Stat 6 signaling.  Ref: 12521997 Blood, 2003
- CD40KO mice primed with C26 GM OX40L  [ **TNFSF4** ]  cells failed to mount a CTL response, and T cells infiltrating the C26 GM OX40L  [ **TNFSF4** ]  tumor were OX40  [ **TNFRSF4** ]  negative, suggesting an impairment in APC T cell cross talk in CD40KO mice.  Ref: 12496388 J Immunol, 2003
- Interaction of OX40  [ **TNFRSF4** ]  CD134  [ **TNFRSF4** ]  on T cells with its ligand OX40L  [ **TNFSF4** ]  on antigen presenting cells has been implicated in pathogenic T cell activation.  Ref: 12631559 Am J Physiol Gastrointest Liver Physiol, 2003
- To examine the in vivo effect of constitutive OX40  [ **TNFRSF4** ]  OX40L  [ **TNFSF4** ]  interaction during immune regulation, we report the establishment of OX40L  [ **TNFSF4** ]  transgenic OX40L  [ **TNFSF4** ]  Tg mice that constitutively express OX40L  [ **TNFSF4** ]  on T cells.  Ref: 12370402 J Immunol, 2002
- The OX 40  [ **TNFRSF4** ]  receptor OX 40R is a member of the tumor necrosis factor  [ **TNFSF4** ]  receptor TNF R superfamily that is expressed on activated CD4 T cells.  Ref: 11518468 Breast Cancer Res Treat, 2001
- In addition, OX40  [ **TNFRSF4** ]  IgG1 protein induces ADCC and complement lysis in OX40  [ **TNFRSF4** ]  ligand  [ **TNFSF4** ]  expressing cells.  Ref: 12223520 J Leukoc Biol, 2002
- The OX40L  [ **TNFSF4** ]  transfer to OX40  [ **TNFRSF4** ]  negative cells was also observed, indicating an OX40  [ **TNFRSF4** ]  independent pathway of OX40L  [ **TNFSF4** ]  transfer.  Ref: 11441094 J Immunol, 2001
- These results indicate that OX40  [ **TNFRSF4** ]  OX40L  [ **TNFSF4** ]  interaction may play a critical role in the development of RA.  Ref: 11069062 Eur J Immunol, 2000
- Therefore, OX40  [ **TNFRSF4** ]  OX40L  [ **TNFSF4** ]  interaction appears to play a critical role in the development of CIA by enhancing Th1 type autoimmune response.  Ref: 11069062 Eur J Immunol, 2000
- The OX 40  [ **TNFRSF4** ]  receptor OX 40R is a cell surface glycoprotein of the tumor necrosis factor  [ **TNFSF4** ]  receptor family that is expressed primarily on activated CD4 T cells.  Ref: 11034096 Cancer Res, 2000
- RESULTS In both hyperplastic thymus of MG and control thymus, OX40  [ **TNFRSF4** ]  cells were scattered mainly in the medulla with much fewer OX40L  [ **TNFSF4** ]  cells being distributed in the corticomedullary junctions.  Ref: 11071109 Acta Neurol Scand, 2000
- BACKGROUND The CD134  [ **TNFRSF4** ]  CD134L  [ **TNFSF4** ]  interaction provides a strong costimulatory signal that is CD28 independent.  Ref: 12134113 Transplantation, 2002
- These findings indicate that 1 resident CD69 OX40  [ **TNFRSF4** ]  IELs constitute a population of partially activated T cells poised for rapid delivery of effector activity, 2 OX40  [ **TNFRSF4** ]  and OX40L  [ **TNFSF4** ]  expression defines IELs that have undergone recent immune activation, 3 OX40  [ **TNFRSF4** ]  IELs are significantly more efficient CTL than are OX40  [ **TNFRSF4** ]  IELs, and 4 the local OX40  [ **TNFRSF4** ]  OX40L  [ **TNFSF4** ]  system plays a critical role in regulating the magnitude of cytokine responses in the gut epithelium.  Ref: 11739485 J Immunol, 2001
- These results suggest that the OX40  [ **TNFRSF4** ]  OX40L  [ **TNFSF4** ]  system is crucial for induction of the allogeneic T cell response and the OX40  [ **TNFRSF4** ]  OX40L  [ **TNFSF4** ]  system is subsequent to and dependent on CD28 signalling, but is crucial for the end outcome of the human alloreactive T cell response.  Ref: 12757617 Immunology, 2003
- CONCLUSION These results present the first evidence that the CD134  [ **TNFRSF4** ]  CD134L  [ **TNFSF4** ]  interaction is associated with acute allograft rejection.  Ref: 12134113 Transplantation, 2002
- After 2 h coculture of activated CD4 T cell OX40L  [ **TNFSF4** ] , OX40  [ **TNFRSF4** ]  with FLAG peptide tagged OX40L  [ **TNFSF4** ]  OX40L  [ **TNFSF4** ]  flag protein expressing COS 1 cells, the OX40L  [ **TNFSF4** ]  flag protein was detected on the cell surface of the CD4 T cells by both anti OX40L  [ **TNFSF4** ]  and anti FLAG mAbs.  Ref: 11441094 J Immunol, 2001
- These results clearly indicate that gp34  [ **TNFSF4** ]  signaling induces expression of RANTES at both mRNA and protein levels in HUVECs and suggest a possible link between the OX40  [ **TNFRSF4** ]  gp34  [ **TNFSF4** ]  system and RANTES during the process of T cell adhesion to endothelial cells and subsequent extravasation.  Ref: 12161277 Immunol Lett, 2002

**Non-interactive relationship** (e.g. studied together, co-existance, homology, etc.)

- To explore the contribution of OX40  [ **TNFRSF4** ]  OX40L  [ **TNFSF4** ]  interaction to the pathogenesis of RA in vivo, we evaluated the effect of a neutralizing anti OX40L  [ **TNFSF4** ]  monoclonal antibody mAb on the development of collagen induced arthritis CIA in DBA 1 mice as an animal model for RA.  Ref: 11069062 Eur J Immunol, 2000
- OX40  [ **TNFRSF4** ] , OX40L  [ **TNFSF4** ] , CD40L, IL 2R and B7 1 as well as IFN gamma and IL 4 mRNA were minimally expressed in normal spleens, usually began to be expressed at 2 weeks and increased to maximal level 4 8 weeks after NaI water.  Ref: 11221995 J Autoimmun, 2001
- CD134  [ **TNFRSF4** ]  is a member of the TNFR family expressed on activated T cells, whose ligand, CD134L  [ **TNFSF4** ] , is found preferentially on activated B cells.  Ref: 10754303 J Immunol, 2000
- However, OX40  [ **TNFRSF4** ]  and OX40L  [ **TNFSF4** ]  expression were rapidly up regulated on CD8 IELs following CD3 stimulation, indicating that both markers on IELs reflect activation dependent events.  Ref: 11739485 J Immunol, 2001
- In this report, we have investigated the role of the tumor necrosis factor  [ **TNFSF4** ]  receptor family member OX40  [ **TNFRSF4** ]  in allergic inflammation using OX40  [ **TNFRSF4** ]  deficient mice.  Ref: 11157058 J Exp Med, 2001
- Blocking the OX40  [ **TNFRSF4** ]  OX40L  [ **TNFSF4** ]  costimulatory pathway differentially affected disease induction in CD28KO mice as compared with WT controls.  Ref: 11238558 J Clin Invest, 2001
- The addition of CD134  [ **TNFRSF4** ]  transfected cells showed only a slight increase in the number of plasma cells compared with that in the culture without them, indicating that an increased Ig production rate per cell is responsible for the observed enhancing effect of CD134L  [ **TNFSF4** ]  engagement rather than increase in plasma cell generation.  Ref: 10754303 J Immunol, 2000
- Consequences of OX40  [ **TNFRSF4** ]  OX40  [ **TNFRSF4** ]  ligand  [ **TNFSF4** ]  interactions in langerhans cell function enhanced contact hypersensitivity responses in OX40L  [ **TNFSF4** ]  transgenic mice.  Ref: 12555678 Eur J Immunol, 2002
- CD134L  [ **TNFSF4** ]  engagement enhances human B cell Ig production CD154 CD40, CD70 CD27, and CD134  [ **TNFRSF4** ]  CD134L  [ **TNFSF4** ]  interactions coordinately regulate T cell dependent B cell responses.  Ref: 10754303 J Immunol, 2000
- Although freshly isolated CD8 IELs exhibited properties of activated T cells CD69 expression and ex vivo cytotoxicity, virtually all CD8 IELs from normal mice were devoid of other activation associated properties, including a lack of expression of OX40  [ **TNFRSF4** ]  and the ligand for OX40  [ **TNFRSF4** ]  OX40L  [ **TNFSF4** ]  and an absence of intracellular IFN gamma staining.  Ref: 11739485 J Immunol, 2001
- OX40  [ **TNFRSF4** ]  ligand  [ **TNFSF4** ]  OX40L  [ **TNFSF4** ]  and OX40  [ **TNFRSF4** ]  CD134  [ **TNFRSF4** ]  are a pair of cell surface molecules belonging to the TNF TNF receptor family.  Ref: 11069062 Eur J Immunol, 2000
- its ligand, OX40  [ **TNFRSF4** ]  ligand  [ **TNFSF4** ]  OX40L  [ **TNFSF4** ]  is expressed on dendritic cells, B cells, and activated endothelial cells.  Ref: 12521997 Blood, 2003
- In these studies, we examined the effects of OX40  [ **TNFRSF4** ]  ligand  [ **TNFSF4** ]  OX40L  [ **TNFSF4** ]  deficiency on the development of Th2 cells during the Th2 immune response to the intestinal nematode parasite Heligmosomoides polygyrus.  Ref: 12496423 J Immunol, 2003
- Our data suggest that CD134  [ **TNFRSF4** ]  CD134L  [ **TNFSF4** ]  is a critical pathway in alloimmune responses, especially recall primed responses, and is synergistic with CD28 B7 in mediating T cell effector responses during allograft rejection.  Ref: 12626546 J Immunol, 2003
- OX40  [ **TNFRSF4** ]  ligand  [ **TNFSF4** ]  transduced tumor cell vaccine synergizes with GM CSF and requires CD40 Apc signaling to boost the host T cell antitumor response.  Ref: 12496388 J Immunol, 2003
- ... blockade of OX40  [ **TNFRSF4** ]  OX40L  [ **TNFSF4** ]  by anti OX40L  [ **TNFSF4** ]  mAb markedly increased IL 10 production.  Ref: 11739485 J Immunol, 2001
- To determine how OX40  [ **TNFRSF4** ]  OX40L  [ **TNFSF4** ]  interaction affects graft versus host disease GVHD, we used antagonistic anti OX40L  [ **TNFSF4** ]  monoclonal antibody mAb or OX40  [ **TNFRSF4** ]  donor or OX40L  [ **TNFSF4** ]  recipient mice.  Ref: 12521997 Blood, 2003
- OX40  [ **TNFRSF4** ]  expression is observed on activated T cells, while OX40L  [ **TNFSF4** ]  is expressed in antigen presenting cells.  Ref: 11346458 Jpn J Cancer Res, 2001
- Engagement of OX 40R by the OX 40  [ **TNFRSF4** ]  ligand OX 40L  [ **TNFSF4** ]  is known to costimulate the production of cytokines by activated T lymphocytes and to rescue effector T cells from activation induced cell death.  Ref: 11034096 Cancer Res, 2000
- To investigate the role of costimulation of OX40  [ **TNFRSF4** ]  in human immunodeficiency virus type 1 HIV 1 infection by its natural ligand, gp34  [ **TNFSF4** ] , the OX40  [ **TNFRSF4** ]  transfected ACH 2 cell line, ACH 2 OX40  [ **TNFRSF4** ] , chronically infected with HIV 1, was cocultured with paraformaldehyde PFA fixed gp34  [ **TNFSF4** ]  transfected mouse cell line, SV T2 gp34  [ **TNFSF4** ] .  Ref: 11435553 J Virol, 2001
- Critical involvement of OX40  [ **TNFRSF4** ]  ligand  [ **TNFSF4** ]  signals in the T cell priming events during experimental autoimmune encephalomyelitis.  Ref: 11509650 J Immunol, 2001
- In the present study, we report the expression of both OX40L  [ **TNFSF4** ]  and OX40  [ **TNFRSF4** ]  on differentiated LC derived from draining lymph nodes in the FITC sensitized mice.  Ref: 12555678 Eur J Immunol, 2002
- To elucidate the role of human OX40  [ **TNFRSF4** ]  OX40L  [ **TNFSF4** ]  interaction, we examined the expression of gp34  [ **TNFSF4** ]  OX40L  [ **TNFSF4** ]  and OX40  [ **TNFRSF4** ]  in normal human hematopoietic cells by using flow cytometry.  Ref: 11346458 Jpn J Cancer Res, 2001
- OX40  [ **TNFRSF4** ]  CD134  [ **TNFRSF4** ] , a membrane bound member of the tumor necrosis factor  [ **TNFSF4** ]  receptor superfamily, is expressed primarily on activated CD4 T cells.  Ref: 11929124 Trends Immunol, 2002
- The present results orchestrated in this study indicate that OX40  [ **TNFRSF4** ]  OX40L  [ **TNFSF4** ]  interaction may be a vital link in our understanding of T cell mediated organ specific autoimmunity.  Ref: 12370402 J Immunol, 2002
- Unlike dendritic cells, the CD4 CD3 cells have little CD80 or CD86 expression but do express high levels of the TNF ligands, OX40  [ **TNFRSF4** ]  ligand  [ **TNFSF4** ]  and CD30 ligand.  Ref: 12753741 Immunity, 2003
- We recently demonstrated a critical contribution of OX40  [ **TNFRSF4** ]  ligand  [ **TNFSF4** ]  OX40L  [ **TNFSF4** ]  to the development of Th2 mediated experimental leishmaniasis.  Ref: 12672051 Eur J Immunol, 2003
- These results indicate that serial measurement of OX40  [ **TNFRSF4** ]  T cells is useful for predicting the onset as well as the therapeutic response of cGVHD and raise a possibility that the OX40  [ **TNFRSF4** ]  gp34  [ **TNFSF4** ]  system is involved in the pathogenesis of cGVHD.  Ref: 11698307 Blood, 2001
- By RT PCR, intrathyroidal expression of OX40L  [ **TNFSF4** ] , OX40  [ **TNFRSF4** ] , CD40L, IL 2R, CTLA 4 and Igbeta mRNA correlated closely with the SAT severity score.  Ref: 11221995 J Autoimmun, 2001
- To further define the contribution of the OX40  [ **TNFRSF4** ]  OX40L  [ **TNFSF4** ]  system to the MLR, we set up a co culture system of CD4 T cells and allogeneic monocyte derived dendritic cells DCs.  Ref: 12757617 Immunology, 2003
- Protein extract derived from the helminth Schistosoma mansoni induced the development of DC2s that promote the development of Th2 cells via the enhanced expression of OX40  [ **TNFRSF4** ]  ligand  [ **TNFSF4** ] .  Ref: 11823500 J Immunol, 2002
- This study was performed to explore the involvement of OX40  [ **TNFRSF4** ]  OX40L  [ **TNFSF4** ]  in the development of T cell mediated chronic colitis.  Ref: 12631559 Am J Physiol Gastrointest Liver Physiol, 2003
- OX40  [ **TNFRSF4** ]  ligand  [ **TNFSF4** ]  OX40L  [ **TNFSF4** ] , a member of TNF superfamily, is expressed on several APC such as splenic dendritic cells DC and activated B cells.  Ref: 12555678 Eur J Immunol, 2002
- However, B cell costimulation via the OX40L  [ **TNFSF4** ]  OX40  [ **TNFRSF4** ]  pathway that has been implicated in CD4 cell expansion, survival, and Th2 development was required.  Ref: 12668647 J Exp Med, 2003
- OX40  [ **TNFRSF4** ]  ligand  [ **TNFSF4** ]  OX40L  [ **TNFSF4** ]  expressed on APCs, and its receptor, OX40  [ **TNFRSF4** ]  present on activated T cells, are members of the TNF TNFR family, respectively, and have been located at the sites of inflammatory conditions.  Ref: 11509650 J Immunol, 2001
- Furthermore, in OX40L  [ **TNFSF4** ]  Tg mice administrated with superantigen, this constitutive OX40  [ **TNFRSF4** ]  OX40L  [ **TNFSF4** ]  interaction on CD4 T cells completely prevented normal in vivo clonal T cell deletion.  Ref: 12370402 J Immunol, 2002
- These results suggest that in NOD.H 2h4 mice, the OX40L  [ **TNFSF4** ] , OX40  [ **TNFRSF4** ] , CD40L and B7 molecules, which increase in the spleen and thyroid of these mice after receiving NaI water, may play a role in SAT development, implying that one or more of these molecules might be good targets for the prevention or treatment of SAT.  Ref: 11221995 J Autoimmun, 2001
- The present study first shows that OX40  [ **TNFRSF4** ]  gp34  [ **TNFSF4** ]  interaction stimulates HIV 1 expression and suggests that OX40  [ **TNFRSF4** ]  triggering by gp34  [ **TNFSF4** ]  may play an important role in enhancing HIV 1 production in both acutely and latently infected CD4 T cells in vivo.  Ref: 11435553 J Virol, 2001
- Amelioration of experimental autoimmune encephalomyelitis with anti OX40  [ **TNFRSF4** ]  ligand  [ **TNFSF4** ]  monoclonal antibody a critical role for OX40  [ **TNFRSF4** ]  ligand  [ **TNFSF4** ]  in migration, but not development, of pathogenic T cells.  Ref: 11160262 J Immunol, 2001
- We reported previously that OX40  [ **TNFRSF4** ] , a member of the tumor necrosis factor  [ **TNFSF4** ]  receptor family, is expressed constitutively on fresh leukemia lymphoma cells isolated from patients with adult T cell leukemia ATL.  Ref: 12416737 Int J Hematol, 2002
- In contrast, the numbers of OX40  [ **TNFRSF4** ]  cells around the germinal centers GC were significantly greater than those of control thymuses, and some mononuclear cells in GC were OX40L  [ **TNFSF4** ] .  Ref: 11071109 Acta Neurol Scand, 2000
- Likewise, toxin from the extracellular bacterium Vibrio cholerae induced development of DC2s as well via an OX40  [ **TNFRSF4** ]  ligand  [ **TNFSF4** ]  independent, still unknown mechanism.  Ref: 11823500 J Immunol, 2002
- This study looks at whether the expression of OX40  [ **TNFRSF4** ] , a member of the tumor necrosis factor  [ **TNFSF4** ]  receptor family, is related to the development of cGVHD in patients who underwent allogeneic hematopoietic stem cell transplantation.  Ref: 11698307 Blood, 2001
- We found three new variations in the OX40  [ **TNFRSF4** ]  ligand  [ **TNFSF4** ]  OX40L  [ **TNFSF4** ] , CD134L  [ **TNFSF4** ]  gene and its 5 upstream region.  Ref: 11197696 Genes Immun, 2000
- Unlike IELs, activated lymph node T cells did not express OX40L  [ **TNFSF4** ] , thus indicating that OX40  [ **TNFRSF4** ]  OX40L  [ **TNFSF4** ]  communication in the intestinal epithelium is part of a novel CD8 network.  Ref: 11739485 J Immunol, 2001
- In addition, T lymphocytes in synovial fluid and synovial tissue from RA patients expressed OX40  [ **TNFRSF4** ] , while OX40L  [ **TNFSF4** ]  was expressed on sublining cells in synovial tissue.  Ref: 11069062 Eur J Immunol, 2000
- This study suggests a possible function of OX40L  [ **TNFSF4** ]  OX40  [ **TNFRSF4** ] , through T cell T cell interaction, in the reactivation of memory T cells in an autocrine manner, with implications for the pathogenesis of viral infections and neoplasms.  Ref: 11346458 Jpn J Cancer Res, 2001
- We investigated the effect of CD134  [ **TNFRSF4** ]  CD134L  [ **TNFSF4** ]  pathway blockade on allograft rejection in fully MHC mismatched rat cardiac and skin transplantation models.  Ref: 12626546 J Immunol, 2003
- When activated in the presence of leukemic CLL B cells, T cells rapidly up regulate CD30 through an OX40  [ **TNFRSF4** ]  ligand  [ **TNFSF4** ]  and interleukin 4 IL 4 dependent mechanism.  Ref: 11175813 Nat Immunol, 2001
- Expression of gp34  [ **TNFSF4** ]  OX40  [ **TNFRSF4** ]  ligand  [ **TNFSF4** ]  and OX40  [ **TNFRSF4** ]  on human T cell clones.  Ref: 11346458 Jpn J Cancer Res, 2001
- New variations in human OX40  [ **TNFRSF4** ]  ligand  [ **TNFSF4** ]  CD134L  [ **TNFSF4** ]  gene.  Ref: 11197696 Genes Immun, 2000
- Association of the CD134  [ **TNFRSF4** ]  CD134L  [ **TNFSF4** ]  costimulatory pathway with acute rejection of small bowel allograft1.  Ref: 12134113 Transplantation, 2002
- MATERIAL AND METHODS We studied the expression of OX40  [ **TNFRSF4** ] , OX40L  [ **TNFSF4** ] , IL 2Ralpha and HLA DR in the thymic tissues of MG and controls using immunocytochemistry and flowcytometry.  Ref: 11071109 Acta Neurol Scand, 2000
- The Role of the CD134  [ **TNFRSF4** ]  CD134  [ **TNFRSF4** ]  Ligand  [ **TNFSF4** ]  Costimulatory Pathway in Alloimmune Responses In Vivo.  Ref: 12626546 J Immunol, 2003
- Immunohistochemistry and flow cytometry were used to analyze the expression and localization of CD134  [ **TNFRSF4** ]  CD134L  [ **TNFSF4** ] .  Ref: 12134113 Transplantation, 2002
- In this study, we show that engagement of OX40  [ **TNFRSF4** ]  by an OX40L  [ **TNFSF4** ]  Ig fusion protein drives IFN gamma production by CD4 T cells and reduces eosinophilia and C. neoformans burden in the lung.  Ref: 12794142 J Immunol, 2003
- Combined CD134  [ **TNFRSF4** ]  CD134L  [ **TNFSF4** ]  blockade may be an effective treatment to both prevent acute allograft rejection and promote the induction of cells with regulatory capacity.  Ref: 12134113 Transplantation, 2002
- To investigate the roles of the co stimulatory molecules CD30 CD30L, CD27 CD27L, OX40  [ **TNFRSF4** ]  OX40L  [ **TNFSF4** ] , and 4 1BB 4 1BBL, which belong to the tumor necrosis factor  [ **TNFSF4** ]  TNF receptor ligand superfamily, in the development of acute viral myocarditis, the expression of these molecules was first analysed in the hearts of mice with acute myocarditis induced by Coxsackievirus B3 CVB3 in vivo.  Ref: 11745696 J Pathol, 2001
- Here we show that signals from a single dose of an agonistic antibody against OX40  [ **TNFRSF4** ]  CD134  [ **TNFRSF4** ] , a member of the tumor necrosis factor  [ **TNFSF4** ]  family of receptors can break an existing state of tolerance in the CD4 T cell compartment.  Ref: 11479622 Nat Med, 2001
- CD27 and CD134  [ **TNFRSF4** ]  ligand  [ **TNFSF4** ]  CD134L  [ **TNFSF4** ]  are two B cell co receptors for T h cell activation induced ligands i.e.  Ref: 11431417 Int Immunol, 2001
- METHODS CD134  [ **TNFRSF4** ]  CD134L  [ **TNFSF4** ]  costimulation was studied in a rat model of small bowel transplantation.  Ref: 12134113 Transplantation, 2002
- However, cytotoxic T lymphocyte CTL clones specific for Epstein Barr virus EBV transformed autologous lymphoblastic cell lines LCLs induced both OX40  [ **TNFRSF4** ]  and OX40L  [ **TNFSF4** ]  expression after antigen or T cell receptor TCR stimulation.  Ref: 11346458 Jpn J Cancer Res, 2001
- The costimulatory molecule OX40  [ **TNFRSF4** ]  ligand  [ **TNFSF4** ]  OX40L  [ **TNFSF4** ] , a member of the tumor necrosis factor  [ **TNFSF4** ]  superfamily, has been shown to be important in T cell priming and cytokine production.  Ref: 12355440 Eur J Immunol, 2002
- We first demonstrated that anti OX40  [ **TNFRSF4** ]  ligand  [ **TNFSF4** ]  anti OX40L  [ **TNFSF4** ]  monoclonal antibody mAb could markedly suppress the mixed leucocyte reaction MLR of peripheral blood mononuclear cells PBMC.  Ref: 12757617 Immunology, 2003
- To determine whether a cellular vaccine that interacts directly with both APC and T cells in vivo might be superior, we generated C26 carcinoma cells transduced with the T cell costimulatory molecule OX40  [ **TNFRSF4** ]  ligand  [ **TNFSF4** ]  OX40L  [ **TNFSF4** ]  either alone C26 OX40L  [ **TNFSF4** ]  or together with GM CSF C26 GM OX40L  [ **TNFSF4** ] , which is known to activate APC.  Ref: 12496388 J Immunol, 2003
- Critical role for OX40  [ **TNFRSF4** ]  ligand  [ **TNFSF4** ]  in the development of pathogenic Th2 cells in a murine model of asthma.  Ref: 12672051 Eur J Immunol, 2003
- RESULTS CD134  [ **TNFRSF4** ]  and CD134L  [ **TNFSF4** ]  molecules were strongly expressed in acutely rejected small bowel allografts.  Ref: 12134113 Transplantation, 2002
- GVHD inhibition by blockade of the OX40  [ **TNFRSF4** ]  OX40L  [ **TNFSF4** ]  pathway did not require CD28 signaling.  Ref: 12521997 Blood, 2003
- In all these patients, the coculture of ATL cells with MMCE OX40  [ **TNFRSF4** ]  ligand  [ **TNFSF4** ]  gp34  [ **TNFSF4** ] , a stable human gp34  [ **TNFSF4** ]  transfectant of a mouse epithelial cell line, resulted in a decrease in the percentage of apoptotic cells after treatment with anti Fas monoclonal antibody, compared to coculture with MMCE mock controls.  Ref: 12416737 Int J Hematol, 2002
- OBJECTIVES To examine the expression of OX40  [ **TNFRSF4** ] , an activated memory T cell marker, and its ligand OX40L  [ **TNFSF4** ] , a set of molecules for T cell B cell interaction, and other lymphocyte activation markers in the thymuses of myasthenia gravis MG and controls.  Ref: 11071109 Acta Neurol Scand, 2000
- Our present results suggested a pivotal role of OX40  [ **TNFRSF4** ]  OX40L  [ **TNFSF4** ]  in the pathogenesis of T cell mediated chronic colitis.  Ref: 12631559 Am J Physiol Gastrointest Liver Physiol, 2003
- OX40  [ **TNFRSF4** ]  stimulation by gp34  [ **TNFSF4** ]  OX40  [ **TNFRSF4** ]  ligand  [ **TNFSF4** ]  enhances productive human immunodeficiency virus type 1 infection.  Ref: 11435553 J Virol, 2001
- Signaling of gp34  [ **TNFSF4** ]  OX40  [ **TNFRSF4** ]  ligand  [ **TNFSF4** ]  induces vascular endothelial cells to produce a CC chemokine RANTES CCL5.  Ref: 12161277 Immunol Lett, 2002
- The CD134  [ **TNFRSF4** ]  CD134  [ **TNFRSF4** ]  ligand  [ **TNFSF4** ]  CD134L  [ **TNFSF4** ]  costimulatory pathway has been shown to be critical for both T and B cell activation.  Ref: 12626546 J Immunol, 2003
- OX40  [ **TNFRSF4** ]  CD134  [ **TNFRSF4** ]  and its ligand OX40L  [ **TNFSF4** ]  have been implicated in T cell activation and migration.  Ref: 11160262 J Immunol, 2001
- Crucially, the up regulation of OX40L  [ **TNFSF4** ]  on antigen presenting cells and CXCR 5 on T cells are CD40 dependent events and we show that T cells do not migrate to follicles in immunized OX40  [ **TNFRSF4** ]  deficient mice.  Ref: 12538659 J Exp Med, 2003
- These data provide insights as to the mechanisms responsible for OX40  [ **TNFRSF4** ]  OX40L  [ **TNFSF4** ]  regulation of GVHD.  Ref: 12521997 Blood, 2003
- We previously showed that gp34  [ **TNFSF4** ]  OX40  [ **TNFRSF4** ]  ligand  [ **TNFSF4** ]  expressed on vascular endothelial cells is not only involved in adhesion between activated T cells and endothelial cells but also by itself able to transmit intracellular signals leading to expression of c fos and c jun mRNA upon OX40  [ **TNFRSF4** ]  binding.  Ref: 12161277 Immunol Lett, 2002
- The CD134  [ **TNFRSF4** ]  CD134L  [ **TNFSF4** ]  pathway has been studied in inflammatory, autoimmune diseases, and graft versus host disease, but no information exists on the involvement of CD134  [ **TNFRSF4** ]  CD134L  [ **TNFSF4** ]  interactions in solid organ transplantation.  Ref: 12134113 Transplantation, 2002
- Expression of OX40  [ **TNFRSF4** ]  and OX40  [ **TNFRSF4** ]  ligand  [ **TNFSF4** ]  gp34  [ **TNFSF4** ]  in the normal and myasthenic thymus.  Ref: 11071109 Acta Neurol Scand, 2000
